# Supplementary material for: Medication-Related Problems in Older People with Multimorbidity in Catalonia: A Real-World Data Study with 5 Years’ Follow-Up
Source: J Clin Med. 2021 Feb 11;10(4):709. doi: 10.3390/jcm10040709 (PMC7916946; doi:10.3390/jcm10040709)
Supplement: Supplementary file 1 [file jcm-10-00709-s001.pdf]

Table S1. Age-adjusted association between medication-related problems and multimorbidity.

**Medication-Related Problems**	**Age OR (95% CI) (*p*-value)**	**2–4 Diseases Influence, Age Adjusted OR (95% CI) (*p*-value)**	**5–9 diseases Influence, Age Adjusted OR (95% CI) (*p*-value)**	**≥10 diseases Influence, Age Adjusted OR (95% CI) (*p*-value)**	**Age Influence, Multimorbidity Adjusted OR (95% CI) (*p*-value)**
Duplicate therapy	1.01 (1.011–1.014) (<0.001)	3.76 (3.53–4.01) (<0.001)	7.49 (7.04–7.97) (<0.001)	13.21 (12.40–14.09) (<0.001)	1 (0.998–1) (0.76)
Drug-drug interaction	1.03 (1.022–1.030) (<0.001)	3.48 (2.70–4.58) (<0.001)	8.10 (6.33–10.60) (<0.001)	19.85 (15.46–26.03) (<0.001)	1.01 (1.006–1.014) (<0.001)
Contraindicated drugs in CKD	1.08 (1.075–1.077) (<0.001)	59.61 (41.76–89.23) (<0.001)	269.47 (188.92–403.11) (<0.001)	695.72 (487.66–1040.92) (<0.001)	1.06 (1.061–1.063) (<0.001)
Contraindicated drugs in liver disease	0.98 (0.980–0.983) (<0.001)	7.45 (6.37–8.77) (<0.001)	17.51 (15–20.59) (<0.001)	35.30 (30.22–42.57) (<0.001)	0.96 (0.963–0.970) (<0.001)
Potentially inappropriate medication	1.04 (1.040–1.042) (<0.001)	4.25 (4.15–4.36) (<0.001)	12.04 (11.75–12.33) (<0.001)	34.89 (33.83–36) (<0.001)	1.02 (1.023–1.025) (<0.001)
Anticholinergic effect-anticholinergic load score					
1	1 (1.002–1.004) (<0.001)	0.94 (0.89–1) (0.04)	1.24 (1.17–1.31) (<0.001)	1.91 (1.81–2.03) (<0.001)	1 (0.998–1) (0.02)
2	1 (1–1.003) (0.005)	1.12 (1.03–1.21) (0.006)	1.73 (1.60–1.87) (<0.001)	2.93 (2.71–3.17) (<0.001)	1 (0.994–0.997) (<0.001)
3 -5	1 (0.996–1) (0.28)	1.13 (0.98–1.30) (0.08)	2.38 (2.08–2.73) (<0.001)	5.77 (5.04–6.61) (<0.001)	0.99 (0.986–0.990) (<0.001)
≥ 6	0.99 (0.986–0.988) (<0.001)	1.52 (1.17–1.99) (0.002)	3.56 (2.79–4.54) (<0.001)	13.04 (10.04–16.92) (<0.001)	0.95 (0.943–0.964) (<0.001)
Increase in fall risk	1 (1.008–1.010) (<0.001)	0.95 (0.91–1) (0.03)	1.12 (1.07–1.17) (<0.001)	1.65 (1.57–1.73) (<0.001)	1 (1.005–1.007) (<0.001)
Effect on QT interval	1.02 (1.019–1.022) (<0.001)	1.07 (1–1.15) (0.03)	1.53 (1.43–1.64) (<0.001)	2.43 (2.27–2.60) (<0.001)	1.02 (1.014–1.017) (<0.001)

Antiulcer agents without criteria for gastroprotection	1.03 (1.029–1.031) (<0.001)	1.18 (1.13–1.24) (<0.001)	1.58 (1.50–1.65) (<0.001)	1.95 (1.86–2.04) (<0.001)	1.03 (1.025–1.027) (<0.001)
Other drugs not recommended for older people	1.02 (1.017–1.019) (<0.001)	1.66 (1.57–1.75) (<0.001)	2.48 (2.35–2.62) (<0.001)	3.84 (3.64–4.06) (<0.001)	1.01 (1.011–1.013) (<0.001)
Patients needing gastroprotection	0.99 (0.978–0.996) (0.004)	1.06 (0.71–1.67) (0.79)	1.04 (0.70–1.63) (0.85)	0.81 (0.54–1.30) (0.36)	0.99 (0.980–0.997) (0.01)

CKD: chronic kidney disease.

Table S2. Number of contraindicated drugs in chronic kidney disease and liver disease and potentially inappropriate medications in older people by multimorbidity group at the end of the study, 2016 (N = 723,016).

Medication-Related Problems	Number of Drugs†	N Comorbidities			Total N = 723,016 n (%)
		2–4 Diseases N = 137,799 n (%)	5–9 Diseases N = 393,672 n (%)	≥10 Diseases N = 191,545 n (%)	
Contraindicated drugs in CKD, n (%)	0	132,600 (96.23%)	330,864 (84.05%)	125,583 (65.56%)	589,047 (81.47%)
	1	3,988 (2.89%)	37,687 (9.57%)	32,006 (16.71%)	73,681 (10.19%)
	2	964 (0.70%)	17,557 (4.46%)	20,959 (10.94%)	39,480 (5.46%)
	3	203 (0.15%)	5,891 (1.50%)	9,282 (4.85%)	15,376 (2.13%)
	4	35 (0.03%)	1,371 (0.35%)	2,833 (1.48%)	4,239 (0.59%)
	5	6 (0.00%)	252 (0.06%)	682 (0.36%)	940 (0.13%)
	6	3 (0.00%)	44 (0.01%)	165 (0.09%)	212 (0.03%)
	7	0 (0.00%)	6 (0.00%)	29 (0.02%)	35 (0.00%)
	8	0 (0.00%)	0 (0.00%)	5 (0.00%)	5 (0.00%)
	11	0 (0.00%)	0 (0.00%)	1 (0.00%)	1 (0.00%)
Contraindicated drugs in liver disease, n (%)	0	135,304 (98.19%)	375,713 (95.44%)	173,755 (90.71%)	684,772 (94.71%)
	1	1,594 (1.16%)	8,761 (2.23%)	6,639 (3.47%)	16,994 (2.35%)
	2	701 (0.51%)	5,496 (1.40%)	5,256 (2.74%)	11,453 (1.58%)
	3	172 (0.12%)	2,695 (0.68%)	3,710 (1.94%)	6,577 (0.91%)
	4	22 (0.02%)	783 (0.20%)	1,540 (0.80%)	2,345 (0.32%)
	5	5 (0.00%)	178 (0.05%)	500 (0.26%)	683 (0.09%)
	6	1 (0.00%)	36 (0.01%)	128 (0.07%)	165 (0.02%)
	7	0 (0.00%)	8 (0.00%)	12 (0.01%)	20 (0.00%)
	8	0 (0.00%)	2 (0.00%)	5 (0.00%)	7 (0.00%)
Potentially inappropriate medication, n (%)	0	77,446 (56.20%)	111,522 (28.33%)	19,904 (10.39%)	208,872 (28.88%)
	1	31,539 (22.89%)	99,893 (25.37%)	32,684 (17.06%)	164,116 (22.69%)
	2	14,892 (10.81%)	72,962 (18.53%)	36,742 (19.18%)	124,596 (17.23%)
	3	7,114 (5.16%)	45,507 (11.56%)	31,208 (16.29%)	83,829 (11.59%)
	4	3,475 (2.52%)	27,568 (7.00%)	23,677 (12.4%)	54,720 (7.57%)
	5	1,632 (1.18%)	15,895 (4.04%)	17,204 (8.98%)	34,731 (4.80%)
	6	818 (0.59%)	9,061 (2.30%)	11,806 (6.16%)	21,685 (3.00%)
	7	404 (0.29%)	5,171 (1.31%)	7,366 (3.85%)	12,941 (1.79%)
	8	237 (0.17%)	2,888 (0.73%)	4,740 (2.47%)	7,865 (1.09%)
	9	122 (0.09%)	1,509 (0.38%)	2,736 (1.43%)	4,367 (0.60%)
	≥ 10	120 (0.09%)	1,696 (0.43%)	3,478 (1.82%)	5,294 (0.73%)

†Number of drugs according to the fifth levels of the Anatomical Therapeutic Chemical Classification System (ATC); CKD: chronic kidney disease; Note: All variables of the table showed a significant difference ($p<0.001$) between multimorbidity groups.

Table S3. Twenty most contraindicated drugs in chronic kidney disease in older people by multimorbidity group at the end of the study, 2016 (N = 723,016).

ATC Code (Drug), n (%)	N Comorbidities			Total N = 723,016 n (%)
	2–4 Diseases N = 137,799 n (%)	5–9 Diseases N = 393,672 n (%)	≥10 Diseases N = 191,545 n (%)	
A10BA02 (Metformin)	488 (0.35%)	12,990 (3.30%)	17,590 (9.18%)	31,068 (4.30%)
C03AA03 (Hydrochlorothiazide)	930 (0.67%)	10,304 (2.62%)	8,814 (4.60%)	20,048 (2.77%)
C09BA02 (Enalapril and diuretics)	899 (0.65%)	8,505 (2.16%)	6,024 (3.14%)	15,428 (2.13%)
N06AB04 (Citalopram)	180 (0.13%)	4,083 (1.04%)	7,054 (3.68%)	11,317 (1.57%)
C09DA01 (Losartan and diuretics)	465 (0.34%)	5,056 (1.28%)	4,812 (2.51%)	10,333 (1.43%)
A10BB09 (Gliclazide)	152 (0.11%)	3,685 (0.94%)	4,731 (2.47%)	8,568 (1.19%)
N02AX02 (Tramadol)	89 (0.06%)	2,693 (0.68%)	5,243 (2.74%)	8,025 (1.11%)
C09CA03 (Valsartan)	235 (0.17%)	3,222 (0.82%)	3,885 (2.03%)	7,342 (1.02%)
C09DA03 (Valsartan and diuretics)	290 (0.21%)	3,227 (0.82%)	2,989 (1.56%)	6,506 (0.90%)
C03DA01 (Spironolactone)	62 (0.04%)	1,804 (0.46%)	4,409 (2.30%)	6,275 (0.87%)
C08CA11 (Manidipine)	201 (0.15%)	2,740 (0.70%)	3,241 (1.69%)	6,182 (0.86%)
M05BA04 (Alendronic acid)	102 (0.07%)	1,961 (0.50%)	3,606 (1.88%)	5,669 (0.78%)
C09BA03 (Lisinopril and diuretics)	256 (0.19%)	2,605 (0.66%)	2,011 (1.05%)	4,872 (0.67%)
M01AE01 (Ibuprofen)	172 (0.12%)	1,725 (0.44%)	1,467 (0.77%)	3,364 (0.47%)
C10AB05 (Fenofibrate)	84 (0.06%)	1,434 (0.36%)	1,652 (0.86%)	3,170 (0.44%)
A10BD07 (Metformin and sitagliptin)	42 (0.03%)	1,287 (0.33%)	1,649 (0.86%)	2,978 (0.41%)
C03BA11 (Indapamide)	149 (0.11%)	1,341 (0.34%)	1,052 (0.55%)	2,542 (0.35%)
C08CA13 (Lercanidipine)	71 (0.05%)	1,163 (0.30%)	1,233 (0.64%)	2,467 (0.34%)
C03EA01 (Hydrochlorothiazide and potassium-sparing agents)	97 (0.07%)	1,279 (0.32%)	1,015 (0.53%)	2,391 (0.33%)
C09CA08 (Olmesartan medoxomil)	91 (0.07%)	1,047 (0.27%)	1,086 (0.57%)	2,224 (0.31%)

ATC: Anatomical Therapeutic Chemical Classification System.

Table S4. Twenty most contraindicated drugs in liver disease in older people by multimorbidity group at the end of the study, 2016 (N = 723,016).

ATC Code (Drug), n (%)	N Comorbidities			Total N = 723,016 n (%)
	2-4 Diseases N = 137,799 n (%)	5-9 Diseases N = 393,672 n (%)	≥10 Diseases N = 191,545 n (%)	
C10AA01 (Simvastatin)	915 (0.66%)	6,558 (1.67%)	6,201 (3.24%)	13,674 (1.89%)
C03CA01 (Furosemide)	114 (0.08%)	2,592 (0.66%)	6,002 (3.13%)	8,708 (1.20%)
A10BA02 (Metformin)	318 (0.23%)	3,430 (0.87%)	3,750 (1.96%)	7,498 (1.04%)
C10AA05 (Atorvastatin)	295 (0.21%)	2,741 (0.70%)	3,366 (1.76%)	6,402 (0.89%)
C03AA03 (Hydrochlorothiazide)	277 (0.20%)	1,920 (0.49%)	1,654 (0.86%)	3,851 (0.53%)
C09CA01 (Losartan)	130 (0.09%)	1,317 (0.33%)	1,736 (0.91%)	3,183 (0.44%)
N05CD06 (Lormetazepam)	107 (0.08%)	1,212 (0.31%)	1,705 (0.89%)	3,024 (0.42%)
C09BA02 (Enalapril and diuretics)	249 (0.18%)	1,572 (0.40%)	1,055 (0.55%)	2,876 (0.40%)
C09DA01 (Losartan and diuretics)	104 (0.08%)	954 (0.24%)	834 (0.44%)	1,892 (0.26%)
C10AA03 (Pravastatin)	73 (0.05%)	721 (0.18%)	1,052 (0.55%)	1,846 (0.26%)
C03CA04 (Torasemide)	59 (0.04%)	688 (0.17%)	936 (0.49%)	1,683 (0.23%)
C09CA03 (Valsartan)	57 (0.04%)	578 (0.15%)	734 (0.38%)	1,369 (0.19%)
C09DA03 (Valsartan and diuretics)	79 (0.06%)	584 (0.15%)	566 (0.30%)	1,229 (0.17%)
A10BX02 (Repaglinide)	17 (0.01%)	362 (0.09%)	670 (0.35%)	1,049 (0.15%)
C08CA11 (Manidipine)	27 (0.02%)	353 (0.09%)	471 (0.25%)	851 (0.12%)
C09BA03 (Lisinopril and diuretics)	79 (0.06%)	434 (0.11%)	311 (0.16%)	824 (0.11%)
A10BD07 (Metformin and sitagliptin)	34 (0.02%)	387 (0.10%)	375 (0.20%)	796 (0.11%)
A05AA02 (Ursodeoxycholic acid)	51 (0.04%)	367 (0.09%)	344 (0.18%)	762 (0.11%)
C10AB05 (Fenofibrate)	26 (0.02%)	313 (0.08%)	331 (0.17%)	670 (0.09%)
N05CF02 (Zolpidem)	27 (0.02%)	243 (0.06%)	312 (0.16%)	582 (0.08%)

ATC: Anatomical Therapeutic Chemical Classification System.

Table S5. Twenty most potentially inappropriate medications in older people by multimorbidity group at the end of the study, 2016 (N = 723,016).

ATC Code (Drug), n (%)	N Comorbidities			Total N = 723,016 n (%)
	2–4 Diseases N = 137,799 n (%)	5–9 Diseases N = 393,672 n (%)	≥10 Diseases N = 191,545 n (%)	
A02BC01 (Omeprazole)	13,838 (10.04%)	88,575 (22.50%)	66,343 (34.64%)	168,756 (23.34%)
N05BA06 (Lorazepam)	10,635 (7.72%)	55,864 (14.19%)	40,603 (21.20%)	107,102 (14.81%)
C07AB07 (Bisoprolol)	6,745 (4.89%)	41,293 (10.49%)	35,910 (18.75%)	83,948 (11.61%)
G04CA02 (Tamsulosin)	5,621 (4.08%)	26,604 (6.76%)	16,487 (8.61%)	48,712 (6.74%)
N05CD06 (Lormetazepam)	3,976 (2.89%)	23,329 (5.93%)	18,190 (9.50%)	45,495 (6.29%)
N02AX52 (Tramadol, combinations)	2,409 (1.75%)	18,022 (4.58%)	14,879 (7.77%)	35,310 (4.88%)
N06AB04 (Citalopram)	2,262 (1.64%)	17,098 (4.34%)	15,382 (8.03%)	34,742 (4.81%)
N06AB06 (Sertralin)	1,794 (1.30%)	14,811 (3.76%)	15,733 (8.21%)	32,338 (4.47%)
N05BA12 (Alprazolam)	2,678 (1.94%)	13,879 (3.53%)	9,757 (5.09%)	26,314 (3.64%)
N02AX02 (Tramadol)	1,193 (0.87%)	10,848 (2.76%)	11,461 (5.98%)	23,502 (3.25%)
C07AB03 (Atenolol)	3,014 (2.19%)	12,977 (3.30%)	6,919 (3.61%)	22,910 (3.17%)
N06AB05 (Paroxetine)	1,802 (1.31%)	11,389 (2.89%)	9,103 (4.75%)	22,294 (3.08%)
N05AH04 (Quetiapine)	1,417 (1.03%)	9,728 (2.47%)	9,275 (4.84%)	20,420 (2.82%)
N05BA01 (Diazepam)	1,800 (1.31%)	9,541 (2.42%)	7,161 (3.74%)	18,502 (2.56%)
N06AX05 (Trazodone)	1,122 (0.81%)	8,180 (2.08%)	8,591 (4.49%)	17,893 (2.47%)
C02CA04 (Doxazosin)	1,505 (1.09%)	8,938 (2.27%)	6,879 (3.59%)	17,322 (2.40%)
C03CA01 (Furosemide)	555 (0.40%)	6,594 (1.67%)	9,899 (5.17%)	17,048 (2.36%)
C07AG02 (Carvedilol)	997 (0.72%)	7,096 (1.80%)	7,399 (3.86%)	15,492 (2.14%)
N02AB03 (Fentanil)	498 (0.36%)	5,926 (1.51%)	8,891 (4.64%)	15,315 (2.12%)
A02BA02 (Ranitidine)	1,287 (0.93%)	7,521 (1.91%)	5,566 (2.91%)	14,374 (1.99%)

ATC: Anatomical Therapeutic Chemical Classification System.
